# Why do people living with HIV not initiate treatment? A systematic review of qualitative evidence from low- and middle-income countries

**DOI:** 10.1016/j.socscimed.2018.05.048

**Published:** 2018-05-30

**Authors:** Shahira Ahmed, Jessica Autrey, Ingrid T. Katz, Matthew P. Fox, Sydney Rosen, Dorina Onoya, Till Bärnighausen, Kenneth H. Mayer, Jacob Bor

**Affiliations:** a Department of Global Health, Boston University School of Public Health, Boston, United States; b Department of Epidemiology, Boston University School of Public Health, Boston, United States; c Health Economics and Epidemiology Research Office, Wits Health Consortium, Faculty of Medical Sciences, University of the Witwatersrand Medical School, Johannesburg, South Africa; d Department of Medicine, Brigham and Women’s Hospital, Boston, MA, United States; e Harvard Medical School, Boston, MA, United States; f Massachusetts General Hospital, Center for Global Health, Boston, MA, United States; g Heidelberg Institute of Public Health, University of Heidelberg, Heidelberg, Germany; h Department of Global Health and Population, Harvard T.H. Chan School of Public Health, Boston, United States; i Africa Health Research Institute, KwaZulu-Natal, South Africa; j The Fenway Institute, Boston, United States

**Keywords:** Qualitative, Systematic review, Antiretroviral therapy, ART, HIV, AIDS, Treatment refusal, LMICs, Care cascade, Continuum of care

## Abstract

**Background:**

Many people living with HIV (PLWH) who are eligible for antiretroviral therapy (ART) do not initiate treatment, leading to excess morbidity, mortality, and viral transmission. As countries move to treat all PLWH at diagnosis, it is critical to understand reasons for non-initiation.

**Methods:**

We conducted a systematic review of the qualitative literature on reasons for ART non-initiation in low- and middle-income countries. We screened 1376 titles, 680 abstracts, and 154 full-text reports of English-language qualitative studies published January 2000–April 2017; 20 met criteria for inclusion. Our analysis involved three steps. First, we used a “thematic synthesis” approach, identifying supply-side (facility) and demand-side (patient) factors commonly cited across different studies and organizing these factors into themes. Second, we conducted a theoretical mapping exercise, developing an explanatory model for patients’ decision-making process to start (or not to start) ART, based on inductive analysis of evidence reviewed. Third, we used this explanatory model to identify opportunities to intervene to increase ART uptake.

**Results:**

Demand-side factors implicated in decisions not to start ART included feeling healthy, low social support, gender norms, HIV stigma, and difficulties translating intentions into actions. Supply-side factors included high care-seeking costs, concerns about confidentiality, low-quality health services, recommended lifestyle changes, and incomplete knowledge of treatment benefits. Developing an explanatory model, which we labeled the Transdisciplinary Model of Health Decision-Making, we posited that contextual factors determine the costs and benefits of ART; patients perceive this context (through cognitive and emotional appraisals) and form an intention whether or not to start; and these intentions may (or may not) be translated into actions. Interventions can target each of these three stages.

**Conclusions:**

Reasons for not starting ART included consistent themes across studies. Future interventions could: (1) provide information on the large health and prevention benefits of ART and the low side effects of current regimens; (2) reduce stigma at the patient and community levels and increase confidentiality where stigma persists; (3) remove lifestyle requirements and support patients in integrating ART into their lives; and (4) alleviate economic burdens of ART. Interventions addressing reasons for non-initiation will be critical to the success of HIV “treat all” strategies.

## 1. Introduction

Many HIV patients in low- and middle-income countries do not immediately start antiretroviral therapy (ART) in spite of being eligible for ART (Bor et al., 2017b, 2018a; Clouse et al., 2013; Fox and Rosen, 2017; Govindasamy et al., 2012; Haber et al., 2017; Kranzer et al., 2012; Mugglin et al., 2012; Rosen and Fox, 2011). In systematic reviews of sub-Saharan African HIV treatment programs, just two-thirds of eligible patients initiated ART (Mugglin et al., 2012; Rosen and Fox, 2011). Uptake of ART is even lower among patients presenting at higher CD4 counts (Bor et al., 2017b, 2018a). As countries implement 2015 World Health Organization (WHO) guidelines to initiate ART at diagnosis regardless of CD4 count (“treat all”), millions of people are newly eligible for ART (World Health Organization, 2016). However, “treat all” will lead to significant increases in ART coverage only if patients diagnosed with HIV go on to start and stay on ART. Patients not yet on ART are at higher risk for morbidity, mortality, and onward transmission of the virus (Bor et al., 2015; Cohen et al., 2011; Danel et al., 2015; Lundgren et al., 2015; Oldenberg et al., 2016; Severe et al., 2010). Given the near-elimination of HIV transmission with virally-suppressive ART, high rates of non-initiation could severely limit the impact of “treat all” strategies.

Despite a large body of evidence of attrition between HIV diagnosis and ART initiation (Bor et al., 2017b, 2018a; Clouse et al., 2013; Fox et al., 2014; Govindasamy et al., 2012; Haber et al., 2017; Kranzer et al., 2012; Mugglin et al., 2012; Rosen and Fox, 2011), less is understood about *why newly* diagnosed patients do not initiate therapy. Prior quantitative reviews have identified predictive risk factors for non-initiation in generalized epidemics in low- and middle-income countries (Govindasamy et al., 2012; Mugglin et al., 2012), including male gender, younger age, lower socioeconomic status, higher transport costs and distance, and perceived stigma. A growing number of qualitative studies have asked patients themselves about their reasons for not initiating ART. Katz et al. (2015) interviewed participants about their reasons for refusing therapy and found that patients made decisions to initiate based on perceived risks of stigma and their subjective views on wellness. Other factors elicited in qualitative studies included lack of social support, fear of disclosure, perceived side-effects, and lack of information on treatment benefits (Unge et al., 2008; Macpherson et al., 2012a,b,c; Katz et al., 2015). Qualitative studies have reported on dimensions that are difficult to quantify, shedding light on the reasons – not just the predictive correlates – of non-initiation and on the mechanisms of patient decision-making.

We conducted a systematic review of the qualitative literature on the reasons why people living with HIV (PLWH) in low- and middle-income countries choose not to start therapy. The review had three objectives. First, we aimed to identify, appraise, and review the qualitative evidence on factors influencing individual-level decisions to initiate or not to initiate treatment, as well as constraints on patients’ choices. Second, we aimed to synthesize this evidence to develop an explanatory model to understand the decision-making process to start (or not to start) ART. Third, we used this model to re-evaluate the review results and to identify opportunities for future interventions to improve ART uptake.

In synthesizing evidence from the qualitative literature, we take an inductive approach, as is frequently used in primary analysis of qualitative data (Creswell, 2013). Rather than fitting our findings to a preselected theory, we reviewed the literature to understand the full range of factors identified and then synthesized these data with elements of several existing models to form an explanatory model for the decision to start, or not to start, ART. Understanding why patients do or do not initiate ART requires explicating complex HIV risk and treatment-seeking behaviors that are influenced by factors at multiple levels (Kaufman et al., 2014). Though individual-level theories have been applied widely to understand HIV prevention and care behavior, these models often focus on specific steps in the individual decision-making process (at the expense of others) (Fisher and Fisher, 2000; Sallis et al., 2015) and have been critiqued for ignoring contextual factors (Kaufman et al., 2014). Other models, such as the socio-ecological model, consider complex multi-level determinants of human behavior (Crosby et al., 2013), but do not specify how these contextual factors are incorporated into a process of decision-making. Integration of models at multiple levels and across disciplines is a promising approach to understand and predict HIV-related health behaviors (Kaufman et al., 2014; Pellowski et al., 2017; Fishbein, 2000).

Our analysis identified several opportunities to intervene to improve patient demand for ART, which go beyond current standard of care in many settings. First, perceptions of the costs and benefits of ART were initially formed during an era when the drugs were toxic and the benefits of early treatment were unknown. Demand for ART could increase if people were better informed about the reduced toxicity profile of current regimens and the health and prevention benefits of early ART. Second, fears of status disclosure in the context of still-rampant HIV stigma discourages many patients from starting ART. Counseling of individual patients to reduce internalized stigma, community-level interventions to reduce externalized stigma and discrimination, and efforts to protect patients from involuntary disclosure could increase ART uptake. Third, many HIV programs require (or at least strongly endorse) a variety of lifestyle changes (e.g., no alcohol, no condom-less sex, disclosure of status), which were cited by several studies as barriers to ART initiation. Treatment uptake would likely increase if these behavioral pre-conditions were replaced with evidence-based harm reduction strategies focused on initiation of and retention on ART, with counseling to support patients in maintaining healthy lifestyles. Finally, starting ART can place significant financial strain on patients and households, and these costs may outweigh the direct benefits of ART for patients early in the course of their HIV infection. Interventions to ease the economic burdens of ART may facilitate wider uptake of therapy. In concert, the above recommendations could help countries achieve higher ART coverage and realize the full population health benefits of HIV “treat all” strategies.

## 2. Method

### 2.1. Systematic review and synthesis of qualitative evidence

We systematically searched for and extracted qualitative evidence on reasons for not starting ART in low- and middle-income countries. We then used qualitative data-analytic techniques to synthesize the data in the included papers (Dixon-Woods and Fitzpatrick, 2001; Thomas and Harden, 2008). The systematic review of qualitative data is gaining recognition as a valid and important approach to “construct evidence from evidence” (Patterson et al., 2001) in order to understand beliefs, attitudes, experiences, and behaviors (Barnett-Page and Thomas, 2009; Dixon-Woods and Fitzpatrick, 2001). Unlike quantitative meta-analyses, designed to produce summary measures of effect, systematic reviews of qualitative data can be used to explore or explain phenomena, e.g., to understand reasons for specific behaviors and to inform the design of interventions (Booth, 2016). Whereas individual qualitative studies face limited generalizability, systematic reviews of qualitative data offer a wider lens, facilitating the development of theory that may be more broadly applicable and therefore more useful in the design of future interventions.

We followed key principles of systematic reviews: the systematic search for studies, assessment of eligibility for inclusion, extraction of data, assessment of study methods, and a pre-specified analytic strategy for data synthesis (see Appendix B for PRISMA checklist) (Liberati et al., 2009). To synthesize the qualitative data, we used “thematic synthesis,” an approach recommended by the Cochrane Qualitative Review Methods Group (Booth et al., 2016; Creswell, 2013; Noyes et al., 2011). Thematic synthesis incorporates elements of both meta-ethnography and grounded theory inasmuch as it is inductive in allowing themes to emerge from the data and to generate new theory (Thomas and Harden, 2008). Thematic synthesis involves two phases: (1) a descriptive phase in which factors are extracted as reported by the authors of the reviewed studies and categories are refined based on the reviewers’ assessment of the data; and (2) an explanatory phase in which connections between different factors are mapped into a theoretical model to explain the phenomenon of interest. We augment this approach by adding (3) an interpretive phase, in which the data are re-assessed in light of the model, providing insight into barriers across the decision-making process and how best to intervene to address them.

### 2.2. Eligibility for inclusion in the review

We reviewed English-language qualitative studies that reported on explanatory factors for why ART-eligible patients initiate, do not initiate, or delay HIV treatment. Studies were selected if the study population included people diagnosed with HIV prior to or during the course of the study and were eligible for ART based on study or country eligibility criteria at the time of the study. ART-eligibility criteria differed across studies.

We included empirical qualitative studies using an array of study designs including ethnographic research, case studies, process evaluations and mixed method designs. Studies were included if they used qualitative methods for data collection such as focus groups, individual interviews, observations, and document analysis, and qualitative methods for data analysis, such as thematic analysis or any other appropriate qualitative analysis method that enabled analysis of text and observations and narrative presentation of findings.

We limited the review to studies from low- and middle-income countries where the population of interest included the general adult population ages 15 years or older to avoid complexity that may be inherent for children in making autonomous healthcare decisions. Studies were excluded if they were restricted to a specific population such as pregnant women, men who have sex with men, sex workers, and people who inject drugs, as reasons for not initiating ART may differ substantively in these populations (Graham et al., 2013; Hodgson et al., 2014; Mehta et al., 2015; Mountain et al., 2014). Studies from high-income countries were also excluded.

### 2.3. Data collection and extraction

#### Search strategy

We searched PubMed, Embase, and Web of Science for eligible studies published between January 1, 2000 and April 30, 2017. We chose January 1, 2000 as start date for our search to ensure that we covered all literature published during the 2000s, the era in which HIV treatment was first scaled up in public sector treatment programs in most low- and middle-income countries (UNAIDS, 2014). Our search terms included combinations of the following words or phrases: HIV, ‘antiretroviral treatment’, refusal, retention, initiation, ‘linkage to care,’ and ‘cascade of care’ (see Appendix A for search terms).

We then searched the reference lists of all systematic reviews on the topic of initiation and retention in the HIV care continuum published within our time frame and we searched the reference lists of all the included manuscripts for relevant titles (see Appendix B for PRISMA checklist).

#### Data extraction and management

We collated studies found from our different search strategies into one database and removed duplicates. Two authors (SA, JA) independently assessed titles and abstracts to determine their potential eligibility and removed studies that were unrelated to the topic of the review. We retrieved the full text of all papers that were likely to be relevant. Two review authors independently reviewed the articles based on the inclusion criteria. Disagreements were resolved through discussion or, when required, with the input of a third reviewer (JB). Reasons for exclusion of articles were recorded. Studies that met review criteria were included in the evidence synthesis. We used a standardized data collection form to extract data from the included studies (see Appendix C for data extraction form). For each study, we extracted information on study population, criteria for ART eligibility, methods for data collection and analysis, key results pertaining to reasons for non-initiation, and authors’ interpretation of these results.

### 2.4. Data analysis

We used a thematic synthesis approach, as described above. Our data analysis consisted of three phases: description of the existing literature, building an explanatory model, and interpreting the findings to identify opportunities for intervention.

#### Description of the existing literature

In the first phase, two review authors independently read and re-read the selected studies and in a first cycle of coding, extracted factors as described by the authors. Factors were grouped into categories defined initially based on our reading of the literature and adjusted iteratively during data extraction. Categories were labeled as demand-side (related to patient demographics and treatment-seeking behaviors) or supply-side (related to health systems capacity and constraints) (Govindasamy et al., 2012; Hontelez et al., 2016) (Table 1). We specifically assessed whether studies analyzed data and presented results separately for men and women.

In a second cycle of coding, we reviewed the factors as described by authors and subjectively grouped factors into sub-categories. In contrast to our first coding cycle, which hewed closely to the authors’ original interpretation of the data, in our second coding cycle, we sought to identify common themes across the studies and to code them according to these themes. We organized our findings into summary tables.

#### Building an explanatory model

In the second phase, we sought to move beyond a descriptive categorization of factors and towards an explanatory model for individuals’ decisions to start (or not to start) ART. We conducted a theoretical mapping exercise to explore the range and nature of factors affecting ART initiation and to identify connections between these factors (Thomas and Harden, 2008). Through a series of discussions by the research team, we mapped these factors into a model of individual decision-making, drawing on both the literature reviewed as well as established models of health behavior. The resulting model offers a theory for how the different factors identified in the review fit together to shape treatment decisions of PLWH.

#### Interpreting the results to identify opportunities for intervention

In the third phase, we re-interpreted the data reviewed in the initial stage of this analysis in light of our explanatory model. We considered how the different factors identified in the review affected patient choice at each stage of the decision-making process. Based on this synthesis, we identified key barriers to ART uptake that future interventions could address.

## 3. Results

### 3.1. Study characteristics and participants

**Fig. 1.**
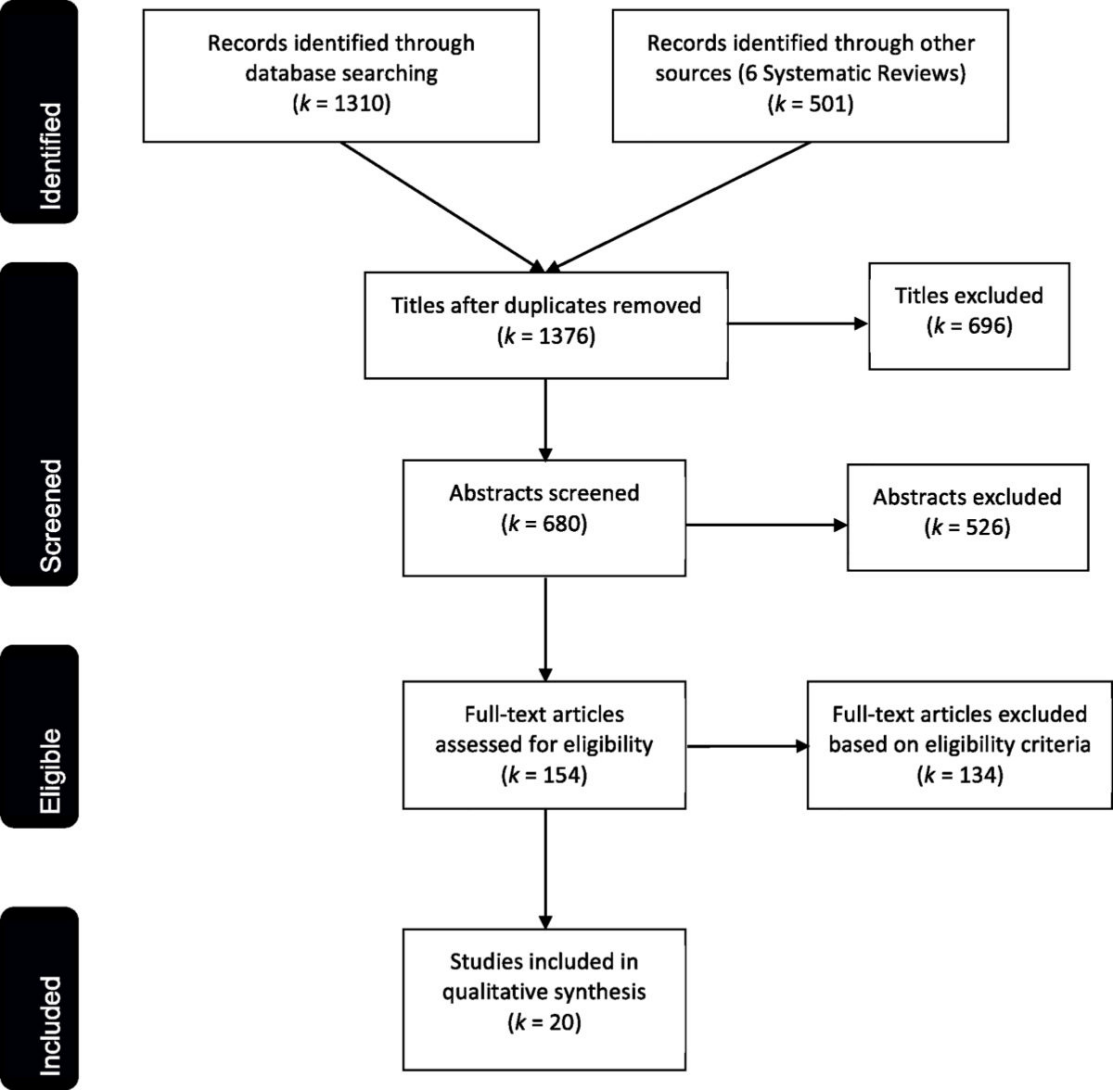
Figure details the numbers of records identified, screened, and included in the review, following PRISMA reporting guidelines.

We reviewed a total of 1376 titles and 680 abstracts from our database searches and from our review of the bibliographies of published systematic reviews and reference lists (Govindasamy et al., 2014; Kranzer et al., 2012; Macpherson et al., 2012a,b,c; Mugglin et al., 2012; Plazy et al., 2015; Rosen and Fox, 2011). At the title and abstract review stages, most articles were excluded because: they were not empirical studies, they were quantitative studies, the study population was from a high-income country, or they focused on specific population groups. We considered 154 full-text papers for inclusion in the review, and 20 met the inclusion criteria (Fig. 1). These 20 articles were included in our descriptive and thematic synthesis (see Appendix D for the list of studies). All of the sampled studies were published between 2006 and 2017, and half of the studies were published in 2013 or later.

All included studies were from sub-Saharan Africa: Ethiopia (1); Ghana (1); Kenya (4); Malawi (2); Mozambique (1); Swaziland (1); South Africa (4); Tanzania (2); Uganda (3); and Zambia (1). All studies included PLWH who accessed health services; additionally some studies included people living with HIV identified in community settings. Most studies also included other types of key informants such as health workers, caregivers, and traditional healers. While all studies documented the number of respondents by gender, most studies reported their results in aggregate, for all patients or for HIV-positive participants, including both men and women. One study described gender differences explicitly for each of their results (Parrott et al., 2011), while another six studies noted gender differences in some of the results or in their discussion (Horter et al., 2017; Kahn et al., 2013; Lambert et al., 2018; Macpherson et al., 2012a,b,c; Musheke et al., 2013; Unge et al., 2008). Our synthesis of the literature thus reflects the limited information on gender-specific factors reported in the included studies.

All but one study used in-depth interviews (IDI) (17 semi-structured IDIs, two structured IDIs) of PLWH, with sample sizes ranging from 8 to 384 participants. Eleven studies also interviewed healthcare providers and other key informants, e.g., community members, care givers, traditional healers (sample sizes ranging from 5 to 52 participants), and ten studies employed Focus Group Discussions (FGD) comprised of patients, healthcare providers and/or other key informants (sample sizes ranging from 4 to 26 FGDs). Two studies reported using direct observation of health facilities. Nineteen of the 20 studies described their approach to data analysis. The studies used the following approaches: ‘thematic analysis’ (9), ‘content analysis’ (6), ‘dimensional analysis’ (1), ‘narrative and case study approach’ (1), ‘framework approach’ (1), and ‘category construction approach to develop an explanatory model’ (1).

### 3.2. Reasons for not starting ART: a thematic synthesis

We summarize below the findings for each of the main categories of factors (representative quotes are presented in Table 2).

#### Socio-demographic and socioeconomic factors

We grouped factors that were related to patient characteristics into this category. A few studies reported gender differences in making decisions about treatment (Abaynew et al., 2011; Kahn et al., 2013; Katz et al., 2015; Macpherson et al., 2012c). For example, one study noted that masculinity influenced men’s feelings of respectability, success, and sexual prowess, which led to their unwillingness to disclose their HIV diagnosis and be seen taking medications (Macpherson et al., 2012c). Another study found that norms regarding femininity shaped some women’s attitudes towards the changes in physical appearance that sometimes occurred with older drug regimens (Katz et al., 2015). Thus, in a context in which ART increases risk of disclosure, gender norms may interact with HIV stigma to discourage patients from starting ART.

In terms of socio-economic status, many studies reported the challenges of poverty and financial burdens, such as transportation costs (distance to the clinic) and food costs as deterring patients from starting treatment (Abaynew et al., 2011; Amuron et al., 2009; Bogart et al., 2013; Katz et al., 2015; N. R. Kunihira et al., 2010; Layer et al., 2014; G. H. Mshana et al., 2006; Musheke et al., 2013; Posse and Baltussen, 2009). Two studies mentioned unstable or short-term housing as factors that were related to patients’ decisions to refuse treatment (Abaynew et al., 2011; Katz et al., 2015). Another study described poor education (illiteracy) as a barrier to raising knowledge and awareness about the benefits of starting treatment (Unge et al., 2008).

#### Health status

The health status of patients at the time of diagnosis was reported in a number of studies as contributing to patients’ decisions to initiate treatment (Abaynew et al., 2011; Amuron et al., 2009; Katz et al., 2015; Layer et al., 2014; Musheke et al., 2013). Three studies found that some patients who felt healthy at the time of diagnosis feared that they would feel worse after taking medication, due to treatment side-effects (Katz et al., 2015; Layer et al., 2014; Musheke et al., 2013). Two studies highlighted the presence of health risk factors (e.g., substance use) that influenced patients’ decision to initiate treatment in the context of guidance from clinical health workers that substance use is incompatible with ART and must be modified prior to initiating therapy (Abaynew et al., 2011; Amuron et al., 2009).

#### Affective factors

Most studies reported on factors that we categorized as related to the emotional or affective state of patients (Abaynew et al., 2011; Amuron et al., 2009; Bogart et al., 2013; Kahn et al., 2013; Katz et al., 2011; Kunihira et al., 2010; Layer et al., 2014; Macpherson et al., 2012a,b,c; Mshana et al., 2006; Muhamadi et al., 2010; Musheke et al., 2013; Parrott et al., 2011; Posse and Baltussen, 2009; Unge et al., 2008; van Loggerenberg et al., 2015; Wachira et al., 2014). In all studies reviewed, the authors mentioned HIV-related stigma as a main barrier affecting patients’ decisions not to initiate treatment. How stigma was defined or conceptualized by investigators varied between studies and was sometimes used as a catchall term for emotions of fear, shame, and guilt. Some studies distinguished “internalized” stigma, the personal assimilation of stigmatizing beliefs leading to feelings of shame and anxiety associated with being HIV positive or on ART (Katz et al., 2013; Turan et al., 2017), from “externalized” stigma, based on participants direct experiences, and “anticipated” stigma, based on participants fears of prejudice and discrimination based on HIV status (Earnshaw et al., 2013a,b; Turan et al., 2017).

Many patients perceived that starting ART would increase the visibility of their HIV status, raising the chances of (or even necessitating) disclosure and putting them at risk of externalized stigma. Indeed, some HIV treatment programs require disclosure to household members before starting ART. Patients feared that being seen at the health facility, being seen taking medications and disclosure could lead to rejection by current partners, peers, family, and community members. A few studies highlighted that women in particular expressed fear of violence and exclusion as a reason for non-initiation (Kahn et al., 2013; Parrott et al., 2011; Unge et al., 2008). Other patients perceived that taking ART would be a constant reminder of their HIV status and cause them distress, consistent with internalized stigma, shame, denial, and non-acceptance of HIV status. It was often difficult to distinguish whether respondents’ fears and emotions were based on their past lived experiences or general perceptions of how others affected by HIV were treated in the community.

Emotions that were not related to stigma but influenced patients’ decisions to refuse treatment included fears and anxiety about clinically-recommended behavior changes that would affect their current way of life and not being mentally prepared for taking on a life-long commitment (Amuron et al., 2009; Kunihira et al., 2010; van Loggerenberg et al., 2015). Perceived constraints on an individual’s way of life appeared to stem not only from the overwhelming list of requirements that are presented to patients, such as routinely going to the clinic and taking medications, but also the requests to modify lifestyle behaviors such as abstaining from alcohol, smoking, and unprotected sex, and disclosing to household members. In addition to overwhelming patients, these requirements appeared to impose upon and to “infantilize” patients, placing moral judgment on their life-style behaviors (Macpherson et al., 2012c). A small number of studies described patients not psychologically ready for treatment, and while this may not have been asked or explored specifically, patients’ general mental health may factor in decisions to initiate or not initiate (Amuron et al., 2009; Kunihira et al., 2010).

A few studies described affective factors that promoted or supported patients’ decisions to initiate treatment (Katz et al., 2015; Parrott et al., 2011; Posse and Baltussen, 2009; van Loggerenberg et al., 2015). These included having coping and resilience strategies and self-determination that allowed patients’ to gain acceptance of their HIV diagnosis. Katz et al. (2015) describe how PLWH who exhibited resilience expressed their “desire to live for others as a reason to overcome concerns about starting treatment,” while others utilized coping skills gained from overcoming other adversity in their lives such as poverty and violence (Katz et al., 2015; Earnshaw et al., 2013a,b).

#### Cognitive factors

Many studies identified factors related to individuals’ knowledge and beliefs about HIV and treatment as key in decisions not to start ART. Several studies reported on patients’ lack of information about the efficacy of ART in improving their health and in reducing transmission (Abaynew et al., 2011; Kahn et al., 2013; Parrott et al., 2011; Posse and Baltussen, 2009). For example, Abaynew et al. (2011) quote one patient as saying: “...I have inadequate knowledge about HIV/AIDS and HIV care. I came to the health facility when I was seriously sick.” Kahn et al. (2013) report that only a few patients interviewed or in focus groups knew that ART can reduce transmission “commenting that the drugs were for the immune system and only a condom could prevent transmission.”

While some patients purported to know of the benefits of ART, studies reported misconceptions about the treatment’s impact on their mortality and morbidity (for example, ART causes cancer) as deterring them from accepting treatment (Kahn et al., 2013; Muhamadi et al., 2010; Musheke et al., 2013; Unge et al., 2008). Additionally, studies frequently reported patients’ fear of ART side-effects, including changes in physical appearance, nausea, and fatigue (Abaynew et al., 2011; Amuron et al., 2009; N. R. Kunihira et al., 2010; Musheke et al., 2013; Unge et al., 2008). Many patients also expressed preference for alternative treatments and methods, including traditional medicine, that they perceived to fit their life better (Katz et al., 2015; Layer et al., 2014; Musheke et al., 2013; Unge et al., 2008). Finally, a few studies described how perceived uncertainty about the future availability of treatment in their community led some patients to weigh the risks of losing access in the future and in turn led to their decision to delay treatment (Kahn et al., 2013; Mshana et al., 2006; Musheke et al., 2013; Unge et al., 2008).

#### Social support

Studies also reported factors related to actual or perceived partner, family, community, and peer support. Studies described the inadequacy of social support as influencing patients’ decisions to start treatment (Abaynew et al., 2011; Kahn et al., 2013; Macpherson et al., 2012c; Muhamadi et al., 2010; Musheke et al., 2013). A few studies linked the lack of emotional support to a fear of disclosure of their status to their social networks, connecting back to the issue of stigma (Amuron et al., 2009). Some studies noted the lack of community education and mobilization efforts as factors that deterred from creating the supportive environment necessary to allow patients to make better decisions about initiating treatment (Abaynew et al., 2011; Kunihira et al., 2010). At least four studies described the positive influence of strong social support from family and friends, leading to acceptance of treatment (Katz et al., 2015; Layer et al., 2014; Parrott et al., 2011; Posse and Baltussen, 2009).

#### Supply-side health system factors

Studies also identified health system and programmatic barriers that deter patients from initiating treatment despite being eligible. In a large number of studies reviewed, participants mentioned concerns regarding quality of care in general or specific terms (Amuron et al., 2009; Bogart et al., 2013; Katz et al., 2015; Kunihira et al., 2010; Layer et al., 2014; Macpherson et al., 2012c; Mshana et al., 2006; Muhamadi et al., 2010; Musheke et al., 2013; Posse and Baltussen, 2009; van Loggerenberg et al., 2015; Wachira et al., 2014). Factors such as inconvenient clinic hours, long queues, difficulty in scheduling appointments, and the prospect of repeated visits to the clinic as influencing patients’ decisions not to start treatment (Bogart et al., 2013; Macpherson et al., 2012c; Posse and Baltussen, 2009; Wachira et al., 2014). Two studies noted rigid and time-consuming service delivery models that did not allow providers to organize delivery of care based on needs and resources at the facility-level (Layer et al., 2014; Macpherson et al., 2012c). Other studies reported fears of breaches of confidentiality and lack of privacy as deterrents for patients (Layer et al., 2014; Muhamadi et al., 2010), consistent with high perceived costs of disclosure. A few studies noted stock-outs in drugs that sometime impeded timely initiation of therapy (Layer et al., 2014; Muhamadi et al., 2010). Finally, several studies reported negative provider-patient interactions as a significant factor in patients’ ability and willingness to initiate treatment (Katz et al., 2015; Layer et al., 2014; Macpherson et al., 2012c; Wachira et al., 2014). Negative past experiences with the health care system and poor reputation of the health facilities within the community also shaped patient perceptions of how they would be treated.

#### Following through on intentions

Some patients said that they did not initiate ART because they feared that they would have difficulty following through on their intentions, specifically around adherence to complex medication regimens (Kahn et al., 2013; Mshana et al., 2006; van Loggerenberg et al., 2015). Additionally, although not directly cited as a reason for non-initiation, some patients may have intended to initiate but not followed through on initiation itself, for similar reasons of procrastination, forgetting, or other factors that make it difficult to translate intentions into actions.

## 4. Discussion

### 4.1. Towards an explanatory model for the decision to start ART

**Fig. 2.**
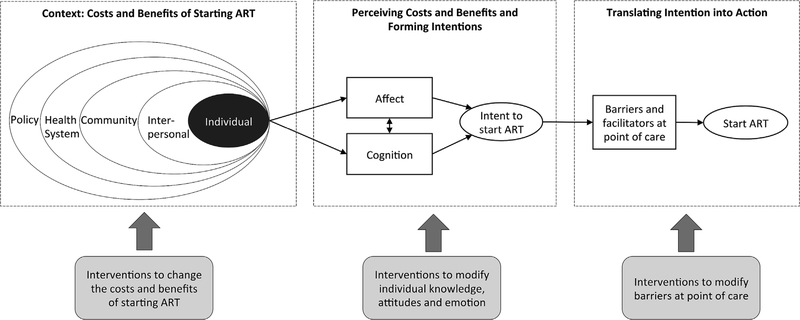
Transdisciplinary Model of Health Decision-Making (TMHD): An Explanatory Model for the Decision to Start ART. Integrating models of health behavior from multiple disciplines, the TMHD model is designed to capture the range of factors shaping the decision to start – or not to start ART – as elucidated by our systematic review of qualitative studies. The decision to start is shaped by (1) the costs and benefits of starting ART, (2) perceptions of those costs and benefits, and (3) the ability to translate intentions into action. The gray arrows indicate that interventions can be designed to modify different stages of this model.

The findings categorized above provide a descriptive analysis of factors found in the literature that influence patients’ decision-making upon learning ART eligibility. Drawing on theory from multiple disciplines, we developed an explanatory model for how these categories are related within the process of patient decision-making (Fig. 2), and labeled this model the Transdisciplinary Model of Health Decision-Making (TMHD). We suggest a three-step process. First, contextual factors at multiple levels determine the costs and benefits of starting therapy, ranging from individual to community to policy and health systems factors. These costs and benefits may be determined by demand-side factors, supply-side factors, or their interaction. These multilevel factors may be modified by patient characteristics – including gender, education, socio-economic status, health status, and social status.

Second, patients perceive their context, forming cognitive and affective appraisals of the costs and benefits of starting ART. They weigh perceived costs and benefits of starting treatment and consider their emotions around starting therapy. Based on these appraisals, patients form intentions to initiate or not to initiate ART.

Third, intentions to start ART may or may not translate into actually starting therapy based on the decision-making context (e.g., how the choice is framed, to what extent patients are encouraged to start, and testing and initiation procedures that remove opportunities for procrastination and support habbit formation). We hypothesize that influencing or intervening at each of these contextual, perceptual, or behavioral points is possible and can influence decisions to start ART when offered to eligible patients.

Our model integrates theory from multiple disciplines to capture different phases of the decision-making process. Following the Social-Ecological model, we situate individual decision-making within a context shaped by multi-level factors, which interact to determine the costs and benefits of starting ART (Reifsnider et al., 2005). As in the Affect-Behavior-Cognition (ABC) model of attitudes (Bakker et al., 2014; Hogg and Vaughan, 2005; Izard, 2010), individuals evaluate costs and benefits through both cognitive appraisals, which may or may not be rational (Kahneman, 2003), and affective appraisals which reflect the emotions (valence, arousal, and motivational intensity) elicited by the considered action. Following the Health Belief Model (Rosenstock, 1974), as well as standard microeconomic theory, individuals form behavioral intentions based on perceived costs and benefits of the action. Finally, following the Theory of Planned Behavior (Ajzen, 1985) as well as much literature in behavioral economics (Camerer et al., 2011; Kahneman, 2003), individuals must translate those intentions into action, a process that may be thwarted by obstacles related to objective circumstances (e.g., additional clinic visits) or human psychology (e.g., procrastination) and which may be facilitated by cues to action (Kaufman et al., 2014) or interventions to assist with behavioral planning and maintenance (Prochaska and Velicer, 1997). The TMHD thus integrates (1) context, (2) the formation of beliefs about that context, and (3) behavioral action on those beliefs, and illustrates how interventions could be implemented at each stage of this process. The three pillars of the TMHD integrate diverse research streams in sociology, health systems studies, neoclassical economics, behavioral economics, and psychology, offering a framework for how research in these disciplines can fit together to understand the upstream/downstream factors in health decision-making.

As with any model, TMHD simplifies behavioral processes for the sake of clarity. For example, TMHD does not explicitly label concepts such as social norms and self-efficacy (Fishbein, 2000), rather modeling these constructs as features of the context and perceived context for decision-making. Self-efficacy – here, whether a person perceives she will have success taking ART – is based on a person’s perceptions of contextual factors related to: how user-friendly the technology is (e.g. single pill vs. multiple pill regimens); whether there are social barriers to use (e.g. stigma leading to fear of disclosure); and behavioral skills shaped by context (e.g. a regular daily schedule; experience taking pills; adherence support; and barriers such as substance use). Another key simplification is the linear nature of the TMHD, which conceals the dynamic nature of decision-making for repeated behaviors such as daily ART. Finally, we note that although not formally portrayed in the TMHD, individual and contextual factors not only modify the objective costs and benefits, but also may modify the ways in which people form beliefs and the ways in which they translate beliefs into action.

### 4.2. Identifying opportunities for future interventions

This systematic review of qualitative studies identified factors implicated in patients’ decisions to initiate or not initiate ART when offered. Despite the remarkable heterogeneity of factors reported in the literature, we were able to categorize the factors into a model that can help explain some of the mechanisms through which decisions are made and that could be amenable to intervention and policy changes.

Thus far, most existing interventions have focused on supply-side factors, developing new drugs and streamlining service delivery in ways that intervene on context to increase the net benefits of ART and facilitate treatment initiation among patients who would like to start (Fox et al., 2016; Govindasamy et al., 2014; MacPherson et al., 2015). For example, simplified drug regimens – one pill, once a day – and drugs with fewer side effects reduce the costs of taking ART and have been shown to increase retention in care (Brennan et al., 2018; Kluberg, 2017). Reduced frequency of drug pickups reduces the time and financial costs of care-seeking (Prust et al., 2017). Reducing the number of required clinic visits between diagnosis and initiation increases progression of patients from intentions to actual uptake of ART (Koenig et al., 2017; Rosen et al., 2016). Based on this evidence, WHO has recently adopted same-day ART initiation among its recommendations for HIV service delivery (World Health Organization, 2017). Our model suggests that while these supply-side interventions address some of the factors that influence decisions to initiate treatment, there are other factors that should be considered in the design of future interventions.

#### Filling the gaps in treatment literacy in communities and the health sector

Although awareness of ART is widespread, the science has evolved, and there are gaps in treatment literacy among patients, providers, and communities. Some patients make decisions not to start treatment based on perceptions of the risks and benefits of ART that are inaccurate, incomplete, or not aligned with the latest evidence. An important reason for these knowledge gaps may be the failure of public health campaigns and HIV counseling practices to update their messaging to reflect the innovations of the last 5–10 years. In particular, information has not fully diffused regarding the lower side effects of the current regimens, regarding the health benefits of early ART (Danel et al., 2015; Lundgren et al., 2015), and regarding the near elimination of HIV transmission for patients on virally-suppressive ART (Cohen et al., 2012; Günthard et al., 2016; Rodger et al., 2016).

The approach to ART scale-up has shifted as the global response has matured, with new rationales for starting therapy (preventing transmission and costly morbidities) and new goals (“treat all”). While the international community has shifted its response to the goals of treating as many people as possible, much treatment knowledge in communities is based on early messaging about the return from sickness to health, rather than preservation of health and prevention of onward transmission. Updating the messaging and information is particularly important for patients who present in better health and who are encouraged to initiate ART before experiencing serious HIV illness. Evidence suggests that it is possible to attain high rates of viral suppression even for patients presenting early in infection (Kwarisiima et al., 2017). Addressing knowledge gaps about the improvements in treatment regimens and the sound reasons for early initiation could be a simple and effective way to increase ART uptake.

#### Addressing stigma at patient and community levels

The findings of this review underscore the implications of stigma for decisions to start ART (Katz et al., 2013; Mak et al., 2017). Persistent HIV stigma discourages patients from starting ART, because taking daily medication forces the patient to come to terms with his/her status and increases the chances of disclosure to others. An effective and comprehensive response to stigma is imperative not only for HIV prevention, but also for the success of treatment and care efforts (Parker and Aggleton, 2003). The findings of this review suggest that efforts to address stigma must involve both short- and long-run strategies. In the long run, HIV stigma needs to be addressed at interpersonal, community, and societal level, with consideration to how gender moderates stigma at each of these levels. Better information about ART may help. There are many foundations for stigmatizing attitudes towards people with HIV, but two key sources are the association of HIV with mortality and the fear of transmission to others. Suppressive ART virtually eliminates HIV-related mortality and HIV transmission. Data we recently collected in rural KwaZulu-Natal, South Africa, reveal that most young adults in the general population are unaware that ART virtually eliminates transmission. Most respondents reported that they would feel uncomfortable having a sexual relationship with someone who is HIV positive even if they are on ART (Bor et al., 2018b). A recent community-randomized trial found that providing information on the prevention benefits of ART lowered stigma and increased participation in HIV testing (Muula et al., 2016). Beyond education about ART, engagement with community leaders to normalize HIV is critical to reduce stigma.

In the short run, stigma can be addressed through health systems interventions that seek to reduce internalized stigma of PLWH and to protect patients from externalized stigma by enabling patients to retain confidentiality. Patients’ internalized feelings of shame due to perceived stigma and discrimination at the time of eligibility need to be countered to support PLWH in making their decisions about treatment. Interventions with PLWH considering therapy should seek actively to de-stigmatize HIV and to improve their skills to address perceived stigma and discrimination. PLWH may worry that starting treatment will lead to forced disclosure if they are seen at HIV clinics and/or have medications around their household. Given the prevalence of stigma in certain contexts, the perceived costs of disclosure may outweigh the benefits of ART even where drugs are available and accessible. In such contexts, particularly for women, promoting patients’ choice to disclose and their right to privacy and confidentiality in seeking treatment could ultimately lead to better programs. For example, the SEARCH trial in Uganda and Kenya sought to reduce patient exposure to stigma by colocating HIV treatment services with other chronic disease services and by training clinic staff to create a friendly, non-judgmental atmosphere (Kwarisiima et al., 2017). In addition to protecting patient privacy and providing support for disclosure, patients should be informed about the costs of not starting treatment: by not taking antiretroviral drugs (ARVs), they will be at increased risk of transmitting HIV to their sex partners and at increased risk of becoming ill, both of which could make disclosure more difficult in the future.

#### Promoting harm-reduction in health systems strategies

Health systems have historically imposed “ordeals” on patients, i.e., hurdles to jump over that ensure that the scarce resources for ART are targeted to patients who are highly motivated to be on therapy (Nichols and Zeckhauser, 1982). Examples include requirements (or strong encouragements) to attend pre-initiation counseling sessions, to disclose to household members, to avoid condom-less sex, and not to drink alcohol or use other substances. Patients who are unwilling to meet these requirements are often deemed “not yet ready” to start life-long therapy. Health systems should assess whether an approach that combines immediate ART with concurrent counseling in these domains would be more effective and avoid screening out many patients who could benefit from ART. Trial evidence suggests that rapid initiation of ART can substantially increase ART uptake with a large proportion of those patients retained in care (Rosen et al., 2016).

To increase ART uptake, the offer of ART should not be contingent upon patients disclosing their status or making lifestyle choices. Further, information on lifestyle choices should be based on rigorous evidence and delivered with care, to avoid adverse unintended consequences. For example, we identified multiple studies where patients chose not to start ART because they were not prepared to give up drinking. Although alcohol dependence may make adherence to daily medication more difficult (Sileo et al., 2016), there is little existing evidence of adverse biological interactions between moderate alcohol use and ART (Bilal et al., 2016; Kalichman et al., 2013; McCance-Katz et al., 2013; Williams et al., 2016). Paradoxically, counseling patients that “ART and alcohol do not mix” may have adverse consequences. In U.S. studies, patients who held this belief were more likely to skip ART doses when they used alcohol, leading to sub-optimal adherence (Kalichman et al., 2013; Sankar et al., 2007).

To take another example, patients starting ART are often counseled that they should disclose to their partners and that they should not have condom-less sex. This guidance may also have unintended ramifications. Although disclosure and condom use are both ideal strategies, they may be impractical for some patients, e.g. people who are not ready to disclose and those who are in relationships where condoms are not always used. If patients choose not to start ART and continue to have condom-less sex with unsuppressed HIV, there is substantial risk of onward transmission. Due to the near elimination of HIV transmission on ART (Bavinton et al., 2017; Rodger et al., 2016), the use of condoms for PLWH who are successfully treated is primarily to prevent STDs and pregnancy. In addition to discouraging PLWH who are not ready to use condoms all the time, insisting on condom use undermines the HIV prevention rationale for ART and may adversely affect demand for treatment.

Finally, counseling protocols should be reviewed to ensure that they do not inadvertently reinforce HIV stigma. In some settings, existing counseling hews closely to the salvation narrative of a person who has sinned (leading to HIV infection) and who now must make lifestyle changes in order to be worthy of life-saving ART. An approach that locates HIV infection in science rather than sin and focuses on harm reduction rather than redemption may help to expand uptake of ART (Hammett et al., 2008; Wolfe et al., 2010).

#### Reducing indirect economic costs to ART initiation

Patients feared that becoming an ART patient would entail economic costs – including transport to the clinic (Bogart et al., 2013; Kunihira et al., 2010) and the need for sufficient food to be able to take the medicines (Singer et al., 2015). The time and transport costs of nominally “free” ART can be very high, as much as a third of household income, in one study in rural South Africa (Chimbindi et al., 2015). Food security has also been identified as a key factor for successful adherence to ART due to the difficulties of taking some regimens on an empty stomach. Some treatment programs counsel patients to ensure that they have access to food before starting ART. In poorer households, the decision to start ART may force difficult decisions about how to prioritize the household food budget (Patenaude et al., 2018). Although ART has substantial benefits to economic productivity and employment recovery in sick patients (Bor et al., 2012; Habyarimana et al., 2010; Larson et al., 2008; Thirumurthy et al., 2008), many patients are now eligible for ART before they have serious symptoms that affect their productivity. The short-run economic benefits of starting ART are more limited early in infection and therefore may be a less significant motivating factor. Addressing demand-side contextual factors related to patients’ socioeconomic circumstances may alleviate the perceived burdens associated with ART and remove a barrier to uptake. For example, a recent RCT in Tanzania found that providing cash incentives and/or food baskets to patients on ART led to large gains in adherence and retention in care (McCoy et al., 2017).

#### Differentiating care to address needs of different groups

Finally, our model posits that multi-level contextual factors influence and modify an individual’s decision to accept or refuse treatment. A person’s characteristics (gender, economic status, and health status) in addition to the social support and social norms they are influenced and bound by affect their decision-making process. These factors imply the need for differentiated treatment pathways that are tailored to ensure demand is improved among different groups to meet the needs of, for example, unmarried women and men of different age groups and of different educational and economic status (Grimsrud et al., 2017, 2016). We found in our review that most qualitative studies did not analyze or present their findings disaggregated by socio-demographic factors such as gender, age, and education, which requires future attention in research to support the design of better interventions and differentiated care models.

### 4.3. Policy implications

In 2015, WHO introduced new guidelines recommending that all patients diagnosed with HIV should be offered treatment (i.e., “treat all”), and several countries have already instituted such policies nationally (Fox and Rosen, 2017; World Health Organization, 2016). By eliminating CD4 criteria, these guidelines remove an important barrier to ART initiation and retention in HIV care (Bor et al., 2017a,b). However, extending eligibility will be insufficient if eligible individuals do not start ART. Our review found that feeling healthy was a key factor in decisions to defer ART, providing an explanation for falling rates of ART uptake at higher CD4 counts in quantitative data (Bor et al., 2018a). With the shift towards “treat all”, more people who feel healthy are going to become eligible for treatment, highlighting the growing importance of understanding and addressing barriers to starting and becoming established on ART. It is possible that reasons for non-initiation will change once people understand that they are eligible for treatment when diagnosed and as more health systems move to offer ART on the same day as diagnosis, per 2017 WHO recommendations. These issues will be of critical importance and need to be explored as countries continue to roll out “treat all”.

### 4.4. Limitations

It is important to acknowledge the limitations of this review. First, we only focused on qualitative research studies and did not include quantitative studies. Whereas quantitative studies can estimate associations with different predictors of non-initiation, qualitative studies reveal the deeper explanations behind these predictors, and can also illuminate factors that are difficult to measure in quantitative surveys or clinical records. For example, male gender is often identified in quantitative studies as a determinant for non-initiation and loss to follow up (Govindasamy et al., 2014; Plazy et al., 2015), which qualitative studies further elucidate as emerging from norms of masculinity (men’s feelings of respectability, success, and sexual prowess) leading to feelings of stigma and fears of disclosure (Macpherson et al., 2012a,b,c). Quantitative studies and reviews have oft noted “clinical factors” such as higher CD4 counts as associated with non-initiation and retention in HIV care (Mugglin et al., 2012; Plazy et al., 2015), which qualitative studies link to fears of side-effects, feeling well, and lack of information about treatment in eliminating transmission (Katz et al., 2015; Layer et al., 2014; Musheke et al., 2013; Rachlis et al., 2016).

Second, there are important limits to the generalizability of our findings. Most of the qualitative studies that we reviewed were based on purposive and convenience samples and were not designed to be representative of an underlying population. For each of these studies, generalizability beyond the participants in these studies is unknown, and our systematic review inherits this limitation. At the same time, qualitative research is designed not to estimate a population parameter, but rather to elucidate explanations and mechanisms for the phenomenon of interest (here, failure to start ART). Though the extent of generalizability is unknown, our systematic review identified recurrent themes across different studies conducted with different participants in different settings, thus raising confidence that these factors may be more broadly relevant to non-initiation of ART than one would consider based on the results from a single qualitative study.

Third, our review only included English peer-reviewed literature and therefore may have missed research conducted in other languages and covering different regions of the world. All of the studies we identified were from Africa. Additionally, we explicitly excluded studies that focused on children, pregnant women, men who have sex with men, sex workers, injecting drug users, or other key populations. Reasons for non-initiation may differ in other settings and populations.

Fourth, our systematic review involved the qualitative analysis of data presented in qualitative papers. Our perspective is necessarily limited to the findings that authors chose to report. Although we followed a clear protocol specified *a priori*, we acknowledge our own subjectivity in assessing and synthesizing the data, a necessary caveat in any qualitative study.

## 5. Conclusions

More patients than ever before are being asked to consider life-long daily HIV therapy, and to do so before they have experienced advanced HIV disease. Designing effective interventions to increase ART uptake will require a better understanding of the factors that influence individuals’ decisions to seek and initiate treatment (Bravo et al., 2010; Keane et al., 2016; MacPherson et al., 2015). Here, the TMHD model can provide guidance. Current interventions have reduced supply-side barriers to initiating ART, making it easier to translate intentions into actions. Supply-side interventions have also reduced the costs of taking ART through the advent of simpler less toxic regimens and differentiated models of care that limit care-seeking costs for stable patients, and they have increased the benefits of ART through enhanced adherence support. However, many patients simply do not want to start ART. Addressing the determinants of patient demand for ART – through interventions that change the context of the treatment decision or patients’ perception of that context – is critical to increasing ART uptake. Understanding the different components of the decision-making process, how they could possibly modify or interact with each other, and where and how to intervene can have a substantial impact on improving uptake of ART and ultimately realizing the full potential of “treat all” policies.

## Supplementary Material

Appendices A-D

## Figures and Tables

**Table 1:** Framework for categorization of factors identified in the literature review.

Dimensions	Definition	Examples
*Demand-side factors for ART Initiation*		
Socio-demographic characteristics	Factors that describe or measure the socio-demographic characteristics of the individual (Kaufman et al., 2014)	Age, gender, marital status
Socio-economic characteristics	Factors that describe or measure the socio-economic characteristics and circumstances of the individual (Kaufman et al., 2014)	Income, education, employment status
Health status	Factors that are related to the self-reported or measured health status of the individual (Kaufman et al., 2014)	CD4 count; feeling healthy; TB co-infection status; other symptoms
Affective factors	Factors that are related to individuals’ affective state We define affect as a psychological term to mean an individual’s expression of emotion or feelings that depends on their emotional state (Izard, 2010). This includes individuals’ moods and emotions, and having feelings of arousal, anxiety, lack of motivation, self-worth (Eagly and Chaiken, 1993; Hogg and Vaughan, 2005)	Fear of stigma; depression
Cognitive factors	Factors that are related mental processes of knowing, thinking and making decisions (Eagly and Chaiken, 1993; Hogg and Vaughan, 2005), which include knowledge, attitudes, and beliefs about HIV and HIV treatment, availability and access to health services, perceptions about health care and providers, and knowledge and beliefs about alternative and traditional treatments	Knowledge of HIV treatment availability; knowledge that HIV treatment can reduce transmission; misconceptions that HIV treatments will kill you
Family/social support	Factors that describe spouse, peer, family or community support (Kaufman et al., 2014)	Presence or absence of social support; support and attitudes of partner
*Supply-side factors for ART initiation*		
Institutional and health systems barriers	Factors related to health care provision, quality of care (Kaufman et al., 2014)	Provider absenteeism; lack of confidentiality; long waiting times; delayed treatment initiation; stock-outs; provider interactions
Financial access	Factors related to financial access of the patient to the health facility (Shengelia et al., 2003)	Costs of care; transport costs
Physical access	Factors related to physical access of the patient to the health facility (Shengelia et al., 2003)	Distance to the clinic
Behavioral barriers and facilitators	Factors related to different ways ART is presented and offered to increase chances that patients will accept ART at point of delivery. This is based on concepts from behavior economics and aspects of “choice architecture” (Thaler et al., 2014)	Reminders, framing, streamlining

**Table 2:** Reasons for not starting ART: representative quotations from studies by category.

Category	Selected quotes	Study citation
Affect	“The things they tell you not to do, like do not eat certain foods and drinks like beer... and cigarettes...they say do not smoke cigarettes. Maybe you like drinking beer, but they say that you should stop. You have to stop it.” *Male participant, Malawi*	MacPherson et al., 2012c
	“If you do not bring your guardian when you are learning you are sent back. They say ‘No. Go and get your guardian and bring her here.’ So, if I did not bring my guardian here, I would not be given the treatment.” *Female participant, Malawi*	MacPherson et al., 2012c
Cognition	“Villagers make others fear to use ARVs. They say they are intended to kill all people with AIDS. Rakai Health Services Program first gave medicine for opportunistic infections only without ARVs. People died, and so they still have that belief that ARVs kill.” *Community member, Uganda*	Kunihira et al., 2010
	“You know what happened is that I had my late sister, second born in our family. Up-to now, I still believe that it is the ARVs that killed her because before she started treatment, yes she used to complain about her health here and there, but when she just started treatment, a week never elapsed and she died, only after 3 or 4 days of starting treatment.” *Male participant, Zambia*	Musheke et al., 2013
	“... I had heard my colleagues at the support group saying that once one started using the ART, one would get a rash, have diarrhoea and have severe headaches or even vomit. Therefore I thought it would be difficult for me to start using the ART.” *Male participant, Kenya*	Unge et al., 2008
	“I don’t use ART. I use the traditional herbs that I am used to, like ‘ingwe’ [an herbal remedy known as an immune booster]. It also tries to kill the virus. It also helps to maintain a healthy life and not lose weight. It boosts the immune system.” *Male participant, South Africa*	Katz et al., 2015
Health Status	“I [feel] very healthy. I found out in 2004 that I am HIV positive [...] I was not understanding negative and positive. They said, ‘You are HIV positive,’ and I said, ‘I am not HIV positive.’ [...] I told myself that I am beautiful and they say I am HIV positive. Are they sick? I said, “No, they are mad. How can they say that?” I am very healthy.” *Female participant, South Africa*	Katz et al., 2015
	“I saw that my body was good and I didn’t have any problem. I was not sick so I decided to stay strong like that without following up on anything.” *Female participant, Tanzania*	Layer et al., 2014
	“For me, I am living a normal life; I am not experiencing any problems. My life is just normal, not until it hits in, you know what I mean, not until it really becomes worse. So, I do not think the medicine will make any difference right now.” *Male participant, 23 years, Zambia*	Musheke et al., 2013
Individual characteristics	“I was a person and I had a good looking body. Even when I was walking people knew that aah that man that is passing there, he is really a man. [...] So I was examining myself and could see that my body was not all right. That is what made me think that aah it’s better to go where I hear that they do some tests...maybe I have a disease. That is why I mustered up boldness to go for testing.” *Male participant, Malawi*	MacPherson et al., 2012c
	“If the service is free, they can come back, but if they need to pay then they have to think about...how they’re going to pay. Some are brought here by their relatives, so they need to rely on those people for them to give them money so they can come back again.” *Counselor, South Africa*	Bogart et al., 2013
	“Drugs are available at treatment centres but these places are far and there are long waiting hours to receive services, high transport costs and walking long distances while we are weak and poor are big barriers to ARVs use.” *Patient, Uganda*	Kunihira et al., 2010
Social Support	“My family and my relatives they all agree because they have seen other people reach the point of being finished [died]. But when they started receiving this treatment they recovered and their bodies become as it was before. So they are the ones who encourage me the most.” *Female participant, Malawi*	MacPherson et al., 2012c
	“HIV positive individuals were highly stigmatized by the community. I didn’t want to disclose my HIV status to others.” *HIV-positive individual, Ethiopia*	Abaynew et al., 2011
	“There was lack of sensitization and awareness regarding the use ARVs; the government thought that routine community health education would work which did not. There should have been special programmes about ARVs.” *Health provider, Uganda*	Kunihira et al., 2010
Health systems factors	“It was troublesome; we used to wake up at 6 a.m. to get [CD4] testing. When you reach there, you find a long line of people and the machine takes only 50 patients, so when you reach 50 it was finished. The others [who did not get tested] had to leave; I had to go there for about a week. I managed to get tested in the second week... You have to wake up about 4 or 5 in the morning so that you can be early; when you are later than that you get turned away.” *Female participant, Tanzania*	Layer et al., 2014
	“Many times there are no ARVs at the centre. Some clients go and they are told they are ready for ARVs but they cannot start because the drugs are not there. We even wait for two to three months when the ARVs are not there. So this is discouraging.” *Male participant, Uganda*	Muhamadi et al., 2010
	“I was on the waiting bench. That’s where they weigh us before we enter into the doctor’s room. That’s when a nurse said, ‘You are wearing tight pants and the way you are seated is seductive and you have even applied eye liner. Who are you trying to attract? You just want to hurt others [i.e., infect others].’ That was so painful... I stopped attending those services [at the regional hospital] because of the statements used in there. I just stayed home because I had already lost hope because of the statements used by some nurses over there. There were very good services until one nurse spoke to me in a very bad way that made me feel worthless, maybe because of the way I am. So I felt really sad. My heart doesn’t feel like going back there because I feel sad every time I see her.” *Female participant, Tanzania*	Layer et al., 2014
	“I would say it is very good and respected because they know our status. We don’t have a problem with them and we are not scared of them. In other places they shout at you in front of everyone. Sometimes people wake up in the early hours of the morning and they have to pay the nurse so that she can give them their files to take the front row. If you don’t pay even if you come in the morning, you can sit on the queue forever. I accompanied my friend and we arrived in the morning, we were told that those who come late have to pay to get their files and if you pay then they put your file on top and the other at the bottom. So there is no respect at all. They have an attitude towards you as if you deliberately choose to be HIV positive.” *Focus group participant, South Africa*	van Loggenerberg et al., 2015
