# Supplementary material for: Why do people living with HIV not initiate treatment? A systematic review of qualitative evidence from low- and middle-income countries
Source: Soc Sci Med. Author manuscript; Available in PMC 2019 Oct 25. (PMC6813776; doi:10.1016/j.socscimed.2018.05.048)
Supplement: Appendices A-D [file NIHMS1054780-supplement-Appendices_A-D.pdf]

Appendix A. Search terms

PUBMED: 1310

HIV Antiretroviral therapy refusal
(("hiv"[MeSH Terms] OR "hiv"[All Fields]) AND antiretroviral[All Fields] AND ("therapy"[Subheading] OR "therapy"[All Fields] OR "therapeutics"[MeSH Terms] OR "therapeutics"[All Fields]) AND refusal[All Fields]) AND (("2000/01/01"[PDAT] : "2017/04/30"[PDAT]) AND English[lang])
243

HIV antiretroviral therapy linkage
(("hiv"[MeSH Terms] OR "hiv"[All Fields]) AND antiretroviral[All Fields] AND ("therapy"[Subheading] OR "therapy"[All Fields] OR "therapeutics"[MeSH Terms] OR "therapeutics"[All Fields]) AND ("genetic linkage"[MeSH Terms] OR ("genetic"[All Fields] AND "linkage"[All Fields]) OR "genetic linkage"[All Fields] OR "linkage"[All Fields])) AND (("2000/01/01"[PDAT] : "2017/04/30"[PDAT]) AND English[lang])
479

HIV antiretroviral therapy cascade
(("hiv"[MeSH Terms] OR "hiv"[All Fields]) AND antiretroviral[All Fields] AND ("therapy"[Subheading] OR "therapy"[All Fields] OR "therapeutics"[MeSH Terms] OR "therapeutics"[All Fields]) AND cascade[All Fields]) AND (("2000/01/01"[PDAT] : "2017/04/30"[PDAT]) AND English[lang])
258

HIV antiretroviral therapy retention initiation
(("hiv"[MeSH Terms] OR "hiv"[All Fields]) AND antiretroviral[All Fields] AND ("therapy"[Subheading] OR "therapy"[All Fields] OR "therapeutics"[MeSH Terms] OR "therapeutics"[All Fields]) AND initiation[All Fields] AND ("retention (psychology)"[MeSH Terms] OR ("retention"[All Fields] AND "(psychology)"[All Fields]) OR "retention (psychology)"[All Fields] OR "retention"[All Fields])) AND (("2000/01/01"[PDAT] : "2017/04/30"[PDAT]) AND English[lang])
330

EMBASE: 946

'human immunodeficiency virus' AND ('antiretroviral therapy'/exp OR art OR initiation) AND 'qualitative research'/exp
243

'human immunodeficiency virus' AND ('antiretroviral therapy'/exp OR art OR 'refusal to participate') AND 'qualitative research'/exp
347

'human immunodeficiency virus' AND ('antiretroviral therapy'/exp OR art OR 'cascade') AND 'qualitative research'/exp
356

Web of Science: 303

TOPIC: (HIV) *AND* **TOPIC:** (antiretroviral therapy) *AND* **TOPIC:** (initiation) *AND* **LANGUAGE:** (English) *AND* **DOCUMENT TYPES:** (Article OR Abstract of Published Item)
Refined by: WEB OF SCIENCE CATEGORIES: (INFECTIOUS DISEASES OR PUBLIC ENVIRONMENTAL OCCUPATIONAL HEALTH) AND [excluding] **COUNTRIES/TERRITORIES:** (USA OR ENGLAND OR JAPAN OR FRANCE OR SWITZERLAND OR CANADA OR AUSTRIA OR SPAIN OR FINLAND OR ITALY OR AUSTRALIA OR NORWAY OR NETHERLANDS OR GERMANY OR IRELAND OR DENMARK OR SCOTLAND OR BELGIUM OR LUXEMBOURG OR GREECE OR ISRAEL OR PORTUGAL) **Timespan:** 2000-2017. **Indexes:** SCI-EXPANDED, SSCI, A&HCI, CPCI-S, CPCI-SSH, BKCI-S, BKCI-SSH, ESCI

Appendix B. PRISMA 2009 Checklist

Section/topic	#	Checklist item	Reported on page #
**TITLE**			
Title	1	Identify the report as a systematic review, meta-analysis, or both.	1
**ABSTRACT**			
Structured summary	2	Provide a structured summary including, as applicable: background; objectives; data sources; study eligibility criteria, participants, and interventions; study appraisal and synthesis methods; results; limitations; conclusions and implications of key findings; systematic review registration number.	2
**INTRODUCTION**			
Rationale	3	Describe the rationale for the review in the context of what is already known.	4
Objectives	4	Provide an explicit statement of questions being addressed with reference to participants, interventions, comparisons, outcomes, and study design (PICOS).	5
**METHODS**			
Protocol and registration	5	Indicate if a review protocol exists, if and where it can be accessed (e.g., Web address), and, if available, provide registration information including registration number.	N/A
Eligibility criteria	6	Specify study characteristics (e.g., PICOS, length of follow-up) and report characteristics (e.g., years considered, language, publication status) used as criteria for eligibility, giving rationale.	6
Information sources	7	Describe all information sources (e.g., databases with dates of coverage, contact with study authors to identify additional studies) in the search and date last searched.	7
Search	8	Present full electronic search strategy for at least one database, including any limits used, such that it could be repeated.	7
Study selection	9	State the process for selecting studies (i.e., screening, eligibility, included in systematic review, and, if applicable, included in the meta-analysis).	7
Data collection process	10	Describe method of data extraction from reports (e.g., piloted forms, independently, in duplicate) and any processes for obtaining and confirming data from investigators.	7

Data items	11	List and define all variables for which data were sought (e.g., PICOS, funding sources) and any assumptions and simplifications made.	8
Risk of bias in individual studies	12	Describe methods used for assessing risk of bias of individual studies (including specification of whether this was done at the study or outcome level), and how this information is to be used in any data synthesis.	N/A
Summary measures	13	State the principal summary measures (e.g., risk ratio, difference in means).	N/A
Synthesis of results	14	Describe the methods of handling data and combining results of studies, if done, including measures of consistency (e.g., I^2) for each meta-analysis.	8

**Section/topic**	**#**	**Checklist item**	**Reported on page #**
Risk of bias across studies	15	Specify any assessment of risk of bias that may affect the cumulative evidence (e.g., publication bias, selective reporting within studies).	N/A
Additional analyses	16	Describe methods of additional analyses (e.g., sensitivity or subgroup analyses, meta-regression), if done, indicating which were pre-specified.	N/A
**RESULTS**			
Study selection	17	Give numbers of studies screened, assessed for eligibility, and included in the review, with reasons for exclusions at each stage, ideally with a flow diagram.	9
Study characteristics	18	For each study, present characteristics for which data were extracted (e.g., study size, PICOS, follow-up period) and provide the citations.	9
Risk of bias within studies	19	Present data on risk of bias of each study and, if available, any outcome level assessment (see item 12).	N/A
Results of individual studies	20	For all outcomes considered (benefits or harms), present, for each study: (a) simple summary data for each intervention group (b) effect estimates and confidence intervals, ideally with a forest plot.	9
Synthesis of results	21	Present results of each meta-analysis done, including confidence intervals and measures of consistency.	10-16
Risk of bias across studies	22	Present results of any assessment of risk of bias across studies (see Item 15).	N/A
Additional analysis	23	Give results of additional analyses, if done (e.g., sensitivity or subgroup analyses, meta-regression [see Item 16]).	16-17
**DISCUSSION**			

Summary of evidence	24	Summarize the main findings including the strength of evidence for each main outcome; consider their relevance to key groups (e.g., healthcare providers, users, and policy makers).	18-23
Limitations	25	Discuss limitations at study and outcome level (e.g., risk of bias), and at review-level (e.g., incomplete retrieval of identified research, reporting bias).	24
Conclusions	26	Provide a general interpretation of the results in the context of other evidence, and implications for future research.	24-25
**FUNDING**			
Funding	27	Describe sources of funding for the systematic review and other support (e.g., supply of data); role of funders for the systematic review.	26

From: Moher D, Liberati A, Tetzlaff J, Altman DG, The PRISMA Group (2009). Preferred Reporting Items for Systematic Reviews and Meta-Analyses: The PRISMA Statement. PLoS Med 6(7): e1000097. doi:10.1371/journal.pmed1000097

Appendix C. Data Extraction Form

NO.	Lead author	Year published	Journal	Title	Country	Study Design	Population	Size	Theory used	ART eligibility criteria	Determinant of ART initiation (reported by study authors)	First-cycle coding			Determinant of ART initiation (summarized by review authors)
												Reviewer 1	Reviewer 2	Final	
1.															
2.															
3.															

Note: Each row represents a single factor or determinant extracted from a study.

Appendix D. Description of studies included in the review (*k* = 20)

Author	Context	Participants	Methods	ART Eligibility Criteria	Notes
Abaynew et al 2011	Ethiopia	Patients and health workers	A case-control study with qualitative component. IDIs with PLWH (8) and providers (10). Data analysis approach: Thematic analysis.	Not stated	Examined factors associated with late presentation to HIV/AIDS care
Amuron et al 2009	Uganda	HIV-infected subjects presenting at the AIDS Support Clinic in Jinja	Observational study with qualitative component. Structured interviews with PLWH (158). Data analysis methods not described.	WHO stage IV, or with CD4 < 200 cell/uL	Assessed reasons patients give when followed-up at home for not returning to complete screening
Ankomah et al 2016	Ghana	Adult HIV-infected persons receiving ART in hospitals; and healthcare providers	A mixed method study: cross-sectional survey and qualitative in-depth interviews. IDIs with providers (4). Data analysis approach: thematic analysis.	Not stated	
Bogart et al 2013	South Africa	Patients and providers at McCord Hospital in Durban	Qualitative study. IDI with patients (10); IDIs with providers (11); FGDs with patients (8). Data analysis approach: thematic analysis.	Not stated	Assessed barriers to linkage to care among people with HIV who were not yet taking antiretroviral treatment
Horter et al 2017	Swaziland	Patients who tested HIV positive; health practitioners; and members of the communities	Qualitative study. IDIs with PLWH (28); IDIs with providers (11). Data analysis approach: thematic analysis.	WHO 2015: Test and treat	

Kahn et al 2013	Kenya	Sub-set of discordant couples participating in longitudinal study	Longitudinal study with qualitative component. IDIs with PLWH (32); FGDs with PLWH (8). Data analysis approach: “Modification of grounded theory called dimensional analysis”.	CD4 < 200 cells/uL; CD4 < 250 cells/uL after Aug 2010	Assessed issues and concerns related to ART initiation among HIV-discordant couples
Katz et al 2015	South Africa	Treatment-eligible adult >18 years, and providers	Qualitative study. IDIs with PLWH (43); IDIs with providers (7). Data analysis approach: “Category construction approach to develop explanatory model”.	CD4 < 350 cells/uL	Examined factors driving refusal of HIV treatment
Kunihira et al 2010	Uganda	People living with HIV and key informants	Qualitative study. IDIs with patients (384); IDIs with key informants (38). Data analysis approach: thematic analysis.	Not stated	Investigated barriers to use of ART in Uganda
Lambert et al 2017	South Africa	Young HIV-positive adults (age 18–35)	Qualitative study. IDIs with PLWH (25). Data analysis approach: content analysis.	WHO 2015: Test and treat	
Layer et al 2014	Tanzania	Patients and providers	Mixed-methods study. IDIs with patients (42); IDIs with providers (52); IDIs with key informants (19); FGDs with key informants and patients (5); direct observations of health facilities (13). Data analysis: “narrative and case-study approach”	WHO stage 3 and 4 , or CD4 < 350 cells/mm3	Explored multi-level barriers and facilitators influencing entry into and engagement in the continuum of care in Iringa, Tanzania
MacPherson et al 2012	Malawi	Health providers and HIV-positive primary care patients both successful and	Qualitative study. IDIs with PLWH (30): IDIs with providers (10). Data analysis approach: framework approach.	WHO stage 3 or 4, or CD4 < 250 cells/uL	Assessed reasons for failure of linkage from HIV testing and counseling to initiation of ART

		unsuccessful in linking to ART			
Mshana et al 2006	Tanzania	Community members and treatment-seekers	Qualitative study. IDIs with patients (18); FGDs with community members (16). Data analysis approach: content analysis.	CD4 count < 200 cells/uL	Part of wider operations research around local introduction of HIV therapy
Muhamadi et al 2010	Uganda	Clients who start ART at 50-200 CD4 counts and those initiated very late at <50 CD4 counts	Qualitative study. IDIs with patients (20); FGDs with care-takers of patients (10). Data analysis approach: content analysis.	CD4 < 350 cells/uL	Explored reasons for late ART initiation among known HIV positive persons in care from a client and caretaker perspective where ART awareness is high
Musheke et al 2013	Zambia	People living with HIV; faith healers; herbal medicine providers; home-based care providers; lay counselors	Qualitative study. IDIs with PLWH (37), ART staff (5), faith healers (5), herbal medicine providers (5), and home-based care providers (5); FGDs with lay HIV counselors (1); direct observations in the community and at an ART clinic. Data analysis approach: "latent content analysis".	Not stated	Using a social ecological framework, investigated barriers to ART initiation in Lusaka, Zambia.
Parrott et al 2011	Malawi	Male and female participants in an ART cohort study at a treatment site	Mixed-methods study. IDIs with patients (60) at hospital then at home; data linked to quantitative clinical and demographic data. Data analysis approach: "open and axial coding of data".	WHO stage 3 and 4, or CD4 < 250 cells/uL	Sought to better understand ART seeking behavior in HIV-infected adults in rural Malawi

Posse et al 2009	Mozambique	People living with HIV and health workers in the districts of Beira and Buzi	Mixed-methods study. Structured interviews (252); IDIs with providers (28); FGDs with patients (13). Data analysis approach: content analysis and factor analysis.	Not stated	Explored factors perceived as barriers to accessing ART
Rachlis et al 2016	Kenya	Patients; caregivers of children with HIV; community leaders; community health workers, other health-care providers	Qualitative study. IDIs with patients and providers (16); FGDs (26). Data analysis approach: thematic analysis using Anderson-Newman Framework for Health Services Utilization.	Not stated	
Unge et al 2008	Kenya	Patients at the Medecins Sans Frontiers (MSF) clinic in the urban Kibera slum	Qualitative study. IDIs with patients (26). Data analysis approach: content analysis.	WHO stage 4; WHO stage 3 & CD4 < 350 cells/uL; or WHO stage 2 & CD4 count <200 cells/uL	Explored why patients offered free ART at the MSF clinic chose not to be treated despite signs of AIDS
Van Loggerenberg 2015	South Africa	Participants in RCT	Qualitative study (sub-set of CAPRISA 058 study). IDIs with patients (30); FGDs with patients (4 Data analysis approach: thematic analysis.	Not clear, but patients included in the study had already initiated ART	Assessed how and why some patients had managed to maintain high levels of adherence, through an analysis of qualitative data on motivations, strategies, and experiences of ART

Wachira et al 2014	Kenya	Patients, community leaders, health workers	Qualitative study. IDIs (16) and FGDs (17) with patients, care-givers, community leaders, providers. Data analysis approach: thematic analysis (described not explicitly stated).	Not clear	Explored perceived health facility barriers to linkage and retention in an HIV care program in western Kenya
